# Assessing suicidality assessments in physician assisted death applications: Do they filter out traditional suicidality?

**DOI:** 10.1192/j.eurpsy.2025.2353

**Published:** 2025-08-26

**Authors:** K. S. Gaind

**Affiliations:** 1 University of Toronto, Toronto, Canada

## Abstract

**Introduction:**

Physician Assisted Death (PAD) has been legalized or decriminalized in over a dozen jurisdictions around the world, and many other jurisdictions are considering assisted dying laws. Most jurisdictions only allow PAD in terminal conditions while a minority allow PAD outside end-of-life situations, with a small number allowing PAD for sole mental illness conditions. A key element in assessments of PAD requests is whether those assessments can filter out traditional suicidality, for which suicide prevention is provided, from other motivations for assisted death, for which PAD may be provided.

**Objectives:**

1. To recognize the range of factors that may motivate assisted dying requests.
2. To understand the factors that inform assisted dying assessments, specifically how the assessments attempt to identify suicidality.
3. To appreciate the degree of certainty or uncertainty that assisted dying assessments actually identify suicidality in different patient populations.

**Methods:**

This presentation briefly reviews evidence related to motivations leading to assisted dying requests in different populations, and then focuses on reviewing guidelines PAD assessors use to attempt to identify traditional suicidality, and to distinguish that from other motivations leading to PAD requests. These guidelines are compared to established evidence and factors related to suicide risk and suicide prevention.

**Results:**

1. Different factors motivate different populations to seek assisted death, with those making PAD requests in terminal situations frequently seeking PAD in efforts to preserve dignity, and those seeking PAD outside terminal conditions or for sole mental illness citing feeling a burden, or an accumulation of multiple life stressors, as fueling their PAD requests.
2. Most of the factors presented in guidance on distinguishing and separating suicidality from PAD requests equally apply to traditionally suicidal individuals and to those requesting PAD.
3. Evidence shows that the few distinguishing factors used to attempt to separate suicidality from PAD requests, specifically impulsivity and acting on one’s own, do not actually filter out suicidality.

**Conclusions:**

While a key goal of physician assisted death assessments is to identify and separate traditionally suicidal individuals from those seeking and receiving assisted death, current assessment techniques are unable to filter out suicidal individuals in an unknown number of cases.

**Disclosure of Interest:**

None Declared

